# GPR30-mediated HMGB1 upregulation in CAFs induces autophagy and tamoxifen resistance in ERα-positive breast cancer cells

**DOI:** 10.18632/aging.203145

**Published:** 2021-06-28

**Authors:** Li Liu, Shengchun Liu, Haojun Luo, Chenxi Chen, Xiaoling Zhang, Lin He, Gang Tu

**Affiliations:** 1 Department of Endocrine and Breast Surgery, The First Affiliated Hospital of Chongqing Medical University, Yu-Zhong 400016, Chongqing, China; 2 Department of Breast and Thyroid Surgery, The Second Affiliated Hospital of Chongqing Medical University, Chongqing 400010, China; 3 Department of Geriatrics, The First Affiliated Hospital of Chongqing Medical University, Yu-Zhong 400016, Chongqing, China; 4 Maternal and Child Care Center Service of Kaizhou, Chongqing 405400, China

**Keywords:** G-protein-coupled estrogen receptor, HMGB1, tamoxifen resistance, cancer-associated fibroblast, autophagy

## Abstract

Tamoxifen (TAM) resistance constitutes a challenge in managing estrogen receptor (ER)α+ breast cancer patients. G-protein-coupled estrogen receptor (GPR30/GPER), which reportedly initiates TAM resistance in ERα+/ GPR30+ breast cancers, is detected in the breast cancer microenvironment, especially cancer associated fibroblasts (CAFs). Herein, considering that GPR30 mediates transcriptional regulation in different cell backgrounds, a microarray strategy was applied in immortalized CAFs derived from primary breast cancer samples, resulting in the identification of 165 GPR30 target genes, among which HMGB1 was confirmed to be upregulated by 17-β estradiol(E2)- and TAM-triggered GPR30 activation in CAFs. Activated GPR30 increased extracellular HMGB1 secretion by CAFs, which was reduced by blocking PI3K/AKT signaling using G15 or LY294002. GPR30-induced HMGB1 upregulation triggered MEK/ERK signaling, leading to increased autophagic behavior to protect cancer cells from TAM-induced apoptosis, mimicking the recombinant HMGB1-mediated increase in cancer cell resistance potential to TAM. MEK/ERK signaling blockage by U0126 decreased the autophagic behavior and resistance ability of cancer cells to TAM. CAF-expressed GPR30 induced TAM resistance via HMGB1 *in vivo*. Overall, TAM upregulated HMGB1 expression and secretion in CAFs via GPR30/PI3K/AKT signaling, and the secreted HMGB1 induced autophagy to enhance TAM resistance in MCF-7 cells in an ERK-dependent manner. Thus, targeting GPR30 and downstream cascades may be an effective strategy to attenuate the resistance of ERα-positive breast tumors to endocrine therapy.

## INTRODUCTION

Based on the etiological role of 17-β-estradiol (E2) in the development and progression of breast cancer, blockade of the E2 signal is utilized as a therapeutic strategy to treat estrogen receptor (ER)-positive breast cancers, which constitutes about 75% of breast cancer cases. Tamoxifen (TAM), a pioneer molecular targeting anticancer agent, is still essential for managing ERα-positive breast cancer. TAM application reduced the risk of recurrence at 5 years by 47 % and the risk of death at 15 years by 34 % in patients with early breast cancer, and prolonged the survival of patients with metastatic breast cancer for about 8 months [1]. However, TAM resistance is the major obstacle in the endocrine therapy of breast cancer. Almost all hormone responsive patients with metastatic tumors and 40 % of those with non-metastatic tumors who receive TAM as an adjuvant therapy eventually experience disease progression, with a deadly outcome [2]. Many molecular pathways, including the alternative or mutated ER signaling, receptor tyrosine kinase signal transduction pathways, phosphatidylinositol 3-kinase-phosphatase and tensin homolog (PI3K-PTEN)/V-Akt murine thymoma viral oncogene homolog (AKT)/mechanistic target of rapamycin (mTOR) pathway, and NF-κB signaling, are reportedly involved in the mechanisms of TAM resistance [3, 4]. However, the underlying mechanisms of TAM resistance are probably multifactorial and remain largely unknown.

Recently, the identification of an alternative ER, G protein coupled estrogen receptor (GPR30), which was identified as a membrane-associated receptor mediating rapid and nongenomic estrogenic effects, including transactivation of epidermal growth factor receptor (EGFR) and production of second messengers, such as cAMP, calcium, and inositol triphosphate in physiological and pathophysiological conditions, has provided a reasonable explanation for TAM resistance in breast cancer for the following reasons. First, activation of GPR30 could transactivate EGFR and downstream signaling, including PI3K, resulting in the promotion of proliferation and migration in breast cancer cell lines [5–7], indicating that GPR30 is a unique receptor, which is linked to multiple factors, as aforementioned, contributing to TAM resistance in breast cancer cells. Second, both TAM and its metabolite, 4-hydroxy-tamoxifen, were demonstrated to be agonists of GPR30, although they are well-known antagonists of ERα [8], indicating the biologic and pharmacologic bases of GPR30-induced TAM resistance. Third, GPR30 was detected in nearly 60 % of breast cancer cases, and coexpression of GPR30 and ERα was confirmed in about 40 % of breast cancer cases [9], indicating the clinical basis of GPR30-induced TAM resistance. Moreover, GPR30 is reportedly a driver of TAM resistance in breast cancer cells coexpressing GPR30 and ERα [10]. In a series of patients with breast cancer with long-term follow-up results, GPR30 was found to be a favorable factor for the outcome of patients, but could be an unfavorable indicator for patients receiving TAM [11, 12].

The tumor microenvironment plays an indispensable role in cancer development, and thus can be recognized as an additional cancer hallmark alongside those that are well established. Cancer associated fibroblasts (CAFs) are known to be the predominant cellular component of the tumor microenvironment, playing a multifaceted role in the development and progression of breast cancer, as they have not only been shown to promote cancer initiation, growth, invasion, metastasis, and therapeutic resistance, but have also been reported to be involved in microenvironmental events, including angiogenesis/lymph angiogenesis, ECM remodeling, cancer-associated inflammation, and metabolism reprogramming [13]. Intriguingly, our group and others have detected GPR30 in breast CAFs [14, 15]. We found that GPR30 was capable of mediating the stimulatory effects of TAM to promote the proliferation and E2 production of CAFs, and was thus assumed that it might contribute to the TAM resistance of cancer cells [14]. Furthermore, the estrogen/GPR30/cAMP/PKA/CREB signaling axis was confirmed to trigger aerobic glycolysis switch in CAFs, introducing a "host-parasite" pattern into tumor cells, thereby causing them to exhibit resistance to several conventional drugs, including Tamoxifen [6]. Together, our research might provide novel insights into the effects of GPER in microenvironment and the induction of TAM resistance.

In our study, we screened GPER-targeted genes by mRNA microarray in breast CAFs and found that high mobility group box 1 (HMGB1) was upregulated. HMGB1 functions as an extracellular signaling molecule and correlated with inflammation, differentiation, cell migration, and tumor metastasis [16]. Although substantial data exists regarding HMGB1 in the setting of apoptosis and necrosis, the role of HMGB1 in autophagy is essentially need to be elucidated. Here, we found that HMGB1secreted by CAFs can induce TAM resistance via MEK/ERK signal pathway also upregulated autophagy in breast cancer cells. These results provide new insights into GPER-mediated CAFs-induced TAM resistance in breast cancers and promising strategy to overcome TAM resistance in clinic.

## RESULTS

### Expression of GPR30 in CAFs was increased in TAM-resistant tumors compared with that in primary tumors

**Figure 1:**
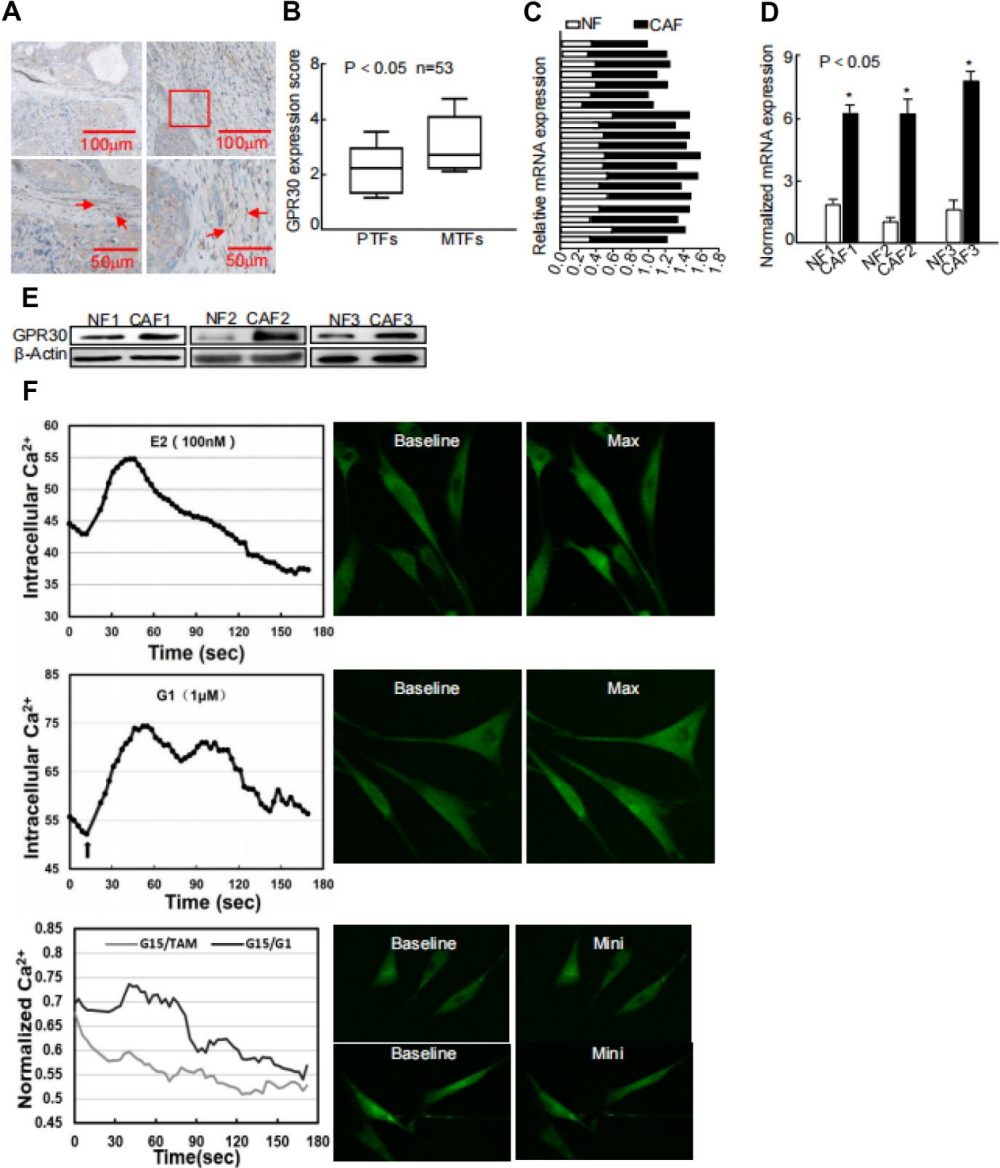
**Abnormally activated and increased GPR30 in TAM-resistant breast CAFs.** (**A**) Representative cases of immunostaining of breast tumor tissue samples, (**B**) Quantitative GPR30 staining in primary tumor fibroblasts (PTFs) and metastatic fibroblasts (MTFs). (**C**) Breast cancer tissues and (**D**) paired immortalized cells evaluated by real time quantitative-PCR. GPR30 was detected in NFs and CAFs; each sample was normalized to its β-actin mRNA content. (*P < 0.05, CAFs vs NFs, Student's *t*-test). (**E**) GPR30 protein content in paired cell lines. (**F**) GPR30 is activated by TAM and G1 in breast CAFs. Treatment with TAM (10 nM) and G1 (1 μM) for 15 min, with or without pretreatment with G15 (1 μM) in CAFs, and monitoring of Ca^2+^ labeled with the Fluo-3/AM probe by laser scanning spectral confocal microscopy. Scale bar: 25 mm (*P < 0.05).

GPR30 was detected in not only the solid parts but also stroma of breast cancer by us and other research groups [15, 17]. Moreover, we observed that GPR30 expression was increased in metastatic and recurrent tumors [7], which may led to TAM resistance, compared with that in paired primary tumors in a series including 53 cases. Intriguingly, this phenomenon was also observed in stromal CAFs (Figure 1A, 1B), indicating that GPR30 might contribute to TAM resistance via both cancer cells and CAFs. To further evaluate the function of GPR30 in CAF-induced TAM resistance, we first detected the mRNA of GPR30 in a series of CAFs and paired normal fibroblasts (NFs), both of which were isolated from primary breast cancer tissues subjected to primary culture. GPR30 expression was 1.41~2.34 fold higher in primary cultured (Figure 1C) and immortalized CAFs (Figure 1D) than in NFs. Correspondingly, higher levels of GPR30 protein were detected in paired CAFs than in NFs (Figure 1E). We next monitored intracellular calcium modulation, which has been shown to serve as a sensor of GPR30 activation [17], in conditions of antagonism with ligands of GPR30. We found that GPR30 was activated in response to TAM and G1 in CAFs, and this stimulation could be blocked by G15, an antagonist of GPR30 (Figure 1F). These data verified the presence of abundant and functional GPR30 protein in CAFs from breast cancer tissues.

### GPR30/PI3K/AKT signaling promoted intracellular expression and extracellular secretion of HMGB1 in mammary CAFs

**Figure 2:**
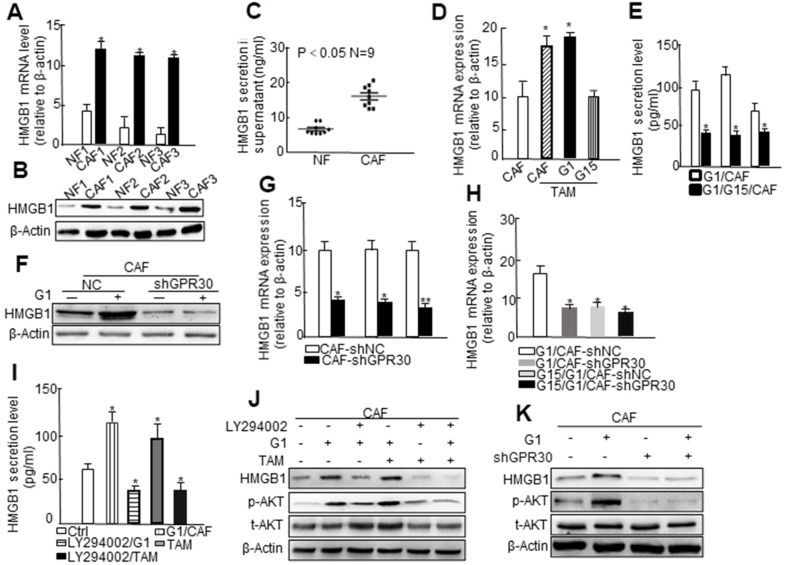
**The PI3K/AKT signaling pathway is involved in GPR30-induced HMGB1 secretion in CAFs.** (**A**) HMGB1 mRNA expression and (**B**) protein levels using paired fibroblasts isolated from immortalized cells and (**C**) HMGB1 secretion in the supernatant of nine immortalized NFs and CAFs. Treatment with G1 (1 μM) and TAM (10 nM) for 30 min, with or without pretreatment with G15 (1 μM) in CAFs, and then detection of HMGB1 mRNA (**D**) and secretion (**E**). All values are shown following normalization against the internal control β-actin (* P < 0.05, ** P < 0.01). Bars represent the mean range of HMGB1 less than 0.05 compared with the matched NF-CM treatment. Detection of the expression of HMGB1 by western blot (**F**) and qRT-PCR (**G**, **H**) after infection with lentivirus carrying GPR30-shRNA. Cells were cultured with CM supplemented with G1 (1 μM) or TAM (10 nM) in the presence or absence of G15 (1 μM) and LY294002 (10 μM) for 24 h, and HMGB1 was detected by ELISA (**I**) and western blotting (**J**). Total Akt and phosphorylated Akt were measured by western blot (**J**, **K**).

GPR30, in addition to ERα/β, mediates the transcriptional effects of estrogens [18]. Therefore, we utilized an mRNA microarray to identify potential target genes of functional GPR30 in mammary CAFs. A total of 165 genes, which were up- or downregulated by administration of both TAM and G1 (Log FC > 2), were selected as target genes of GPR30. We enriched all selected genes and analyzed them using the KEGG database, and found corresponding signaling pathways (e.g. PI3K/AKT, MAPK), which were probably associated with GPR30 (Supplementary Figure 1A). As CAFs are known to significantly contribute to tumor progression in an “afferent” pattern (that is, influence the malignancy of cancer cells by paracrine signaling through growth factors, hormones, and cytokines), we analyzed the presence of genes encoding cytokines among the GPR30-targeted genes in CAFs. Among the selected genes, we verified the presence of a set of genes encoding cytokines, such as high mobility group box 1 (HMGB1), calcium dependent secretion activator (CADPS), and protein detoxification 42 (DTX42) (Supplementary Figure 1B). We selected for further assessment HMGB1, a multifunctional protein that for might be involved in tumor progression and drug resistance through by-pass signaling, and has also been reported to be related to the recurrence free survival (RFS) of patients with breast cancer (Supplementary Figure 1C) [19]. We found that both the mRNA and protein levels of HMGB1 were higher in 3 randomly selected primary CAFs than in paired NFs (Figure 2A, 2B). Moreover, the concentrations of HMGB1 were significantly increased by about 2 times (P<0.05) in supernatants from CAFs compared with those of NFs (Figure 2C). Based on data provided by bioinformatic analysis and the fact that the expression of HMGB1 is associated with that of GPR30 in breast cancer tissues (Supplementary Figure 1D, 1E), we verified the interaction between these two molecules. Immortalized CAFs were treated with G1 (1 μM) or TAM (10 nM) with or without pretreatment with G15 (1μM), and then we detected the expression of HMGB1 in the cell fragment and supernatant. As expected, G1 and TAM stimulated the activation of GPR30 and promoted the mRNA expression and the extracellular secretion of HMGB1, whereas G15 reduced the stimulating effects induced by G1 and TAM (Figure 2D, 2E). In addition, knockdown of GPR30 in CAFs (Figure 2F) notably reduced TAM- and G1-stimulated production of HMGB1 in supernatants (Figure 2G, 2H). Thus, TAM upregulated the expression and extracellular secretion of HMGB1 via the activation of GPR30 in stromal CAFs.

PI3K/AKT signaling is an important pathway downstream of the activation of GPR30 [20]. The aforementioned data (Supplementary Figure 1B) also indicated that the PI3K/AKT pathway might be involved in the regulation of target genes of GPR30, such as HMGB1. To confirm the involvement of PI3K/AKT signaling in the GPR30-induced cellular expression and extracellular secretion of HMGB1 in CAFs, we administered the LY294002-PI3K inhibitor under the aforementioned conditions and found that the secretion (Figure 2I) and protein (Figure 2J) expression of HMGB1 induced by pretreatment with TAM and G1, which were notably attenuated by the LY294002-PI3K inhibitor. Similarly, knockdown of the *GPR30* by short hairpin RNA (shRNA) blocked the TAM- and G1- induced upregulation of HMGB1, and further caused a marked decrease in the phosphorylation of AKT under stimulation with G1 (Figure 2K) and TAM (Supplementary Figure 1F). These data indicated that the GPR30/PI3K/AKT signaling pathway might promote the intracellular expression and extracellular secretion of HMGB1 in mammary CAFs.

### CAFs-derived HMGB1 promoted TAM resistance in ERα-positive breast cancer

**Figure 3:**
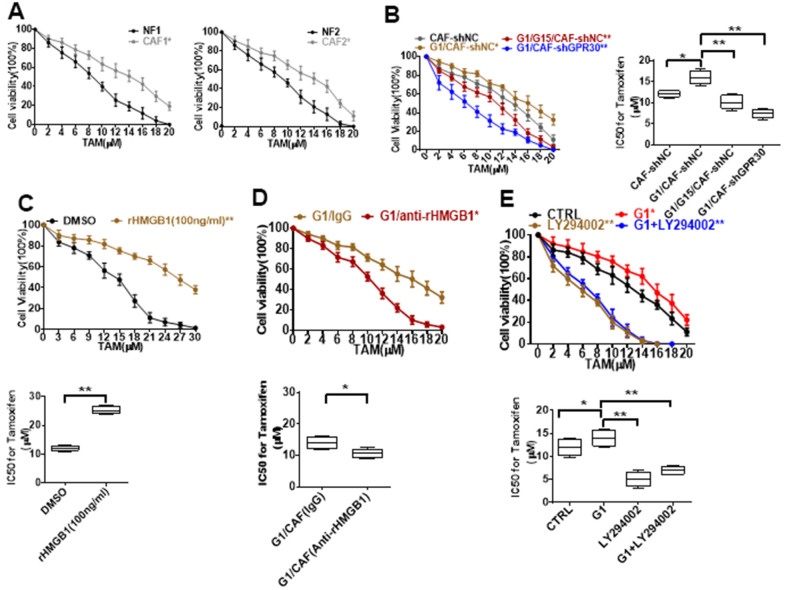
**Resistance to TAM is positively associated with HMGB1 elevation.** (**A**, **B**) CCK8 detection of the viability of MCF-7 cells cultured with CM from immortalized cells and CM from CAF-shGPR30 cells pretreated with G1 or TAM in the presence or absence of G15 for 24 h. *P < 0.05 vs control; ** P < 0.05 vs control; ctrl: MCF-7 cells cocultured with fresh phenol red-free medium. The viability of MCF-7 cancer cells was notably changed with the addition of (**C**) recombinant HMGB1, (**D**) anti-rHMGB1, and (**E**) LY294002. (*P < 0.05 vs control; ** P < 0.01 vs control; ctrl: MCF-7 cells cocultured with fresh phenol red-free medium supplemented with DMSO). All data were analyzed by the IC50 software, and repeated at least 3 times.

CAFs have been shown to be an important driver of cancer progression in the breast cancer microenvironment, including the resistance to endocrine therapy. Thus, we cocultured MCF-7 cells with conditional medium (CM) derived from CAFs or NFs and treated them with TAM for 24 h, and then evaluated cell viability. The CM derived from CAFs endowed MCF-7 cells with a stronger ability for survival under administration of TAM in comparison with CM from NFs (Figure 3A). Meanwhile, the CM derived from G1-treated CAFs further promoted the survival of MCF-7 cells under administration of TAM (Figure 3B). Moreover, pretreating CAFs with G15 or knockdown of GPR30 in CAFs attenuated this pro-survival effect (Figure 3B). Thus, GPR30 protein was considered to be responsible for the CAF-induced TAM resistance in MCF-7 cells.

Moreover, treatment of MCF-7 cells with recombinant human HMGB1 endowed MCF-7 cells with a higher survival ability under exposure to TAM in comparison with control cells (Figure 3C), mimicking the promotive effects of CM derived from CAFs on MCF-7 cells. To further confirm the function of CAF-derived HMGB1 in triggering resistance of MCF-7 cells to TAM, we used a specific neutralizing antibody against HMGB1. As expected, exhaustion of HMGB1 by the neutralizing antibody in the CM derived from CAFs (IC50 = 10.46 ± 1.38) increased the sensitivity of MCF-7 cells to TAM, as indicated by the respective IC50 values (IC50 = 12.09 ± 0.79) (Figure 3D). To further confirm that HMGB1 secreted via the GPR30/PI3K/Akt signaling pathway is key in promoting cell resistance, we pretreated MCF-7 cells with LY294002 before adding TAM, the key pathway inhibitor, finding that the addition of this inhibitor LY294002 significantly reduced the cell viability, implying that the cells are more sensitive to drugs (Figure 3E). These data demonstrate that CAF-derived HMGB1 is indeed involved in the tolerance of ERα-positive (ERα+) breast cancer cells to TAM.

### HMGB1 induced autophagy to enhance TAM resistance via MEK/ERK signaling in ERα-positive breast cancer cells

**Figure 4:**
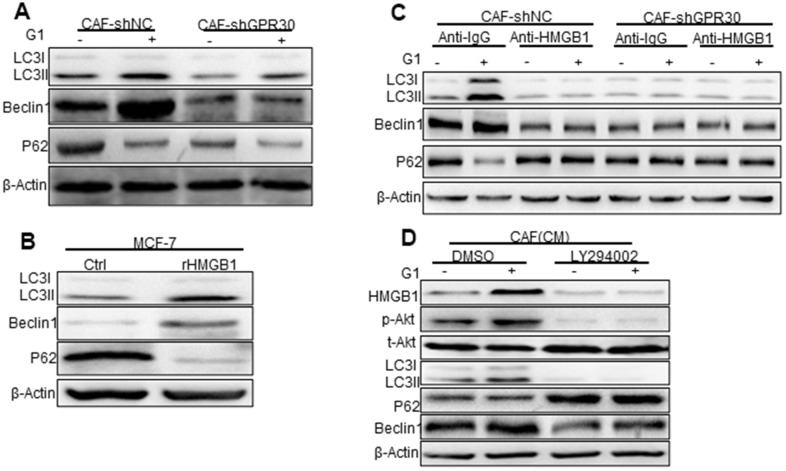
**Phosphorylated Akt is involved in HMGB1-mediated autophagy.** (**A**) The Beclin1, p62, and LC3, autophagy-related marker proteins in MCF-7 cells cultured with different conditioned medium (CM) from CAFs, (**B**) with recombinant HMGB1, or with (**C**) HMGB1 neutralizing antibody detected by western blotting. (Control: MCF-7 cells cultured with DMEM phenol red-free medium; CAF-shNC (CM): MCF-7 cells cultured with CM from CAF-shNC cells; G1+CAF-sh NC (CM): MCF-7 cells cultured with CM from CAF-shNC cells treated with G1; CAF-shGPR30 (CM): MCF-7 cells cultured with CM from CAF-shGPR30 cells; G1+CAF-sh GPR30 (CM): MCF-7 cells cultured with CM from CAF-shGPR30 cells treated with G1. *P < 0.05, vs CAF(CM) and CAF-shNC (CM **P < 0.01, vs CAF(CM) and CAF-shNC (CM); ∆P < 0.01, G1+CAF-shNC (CM) (n = 3). (**D**) Phosphorylated Akt was detected with the addition of LY294002 by western blot analysis. All experiments were independently repeated at least 3 times.

Previous studies have linked HMGB1 to the process of autophagy [21, 22], which is known to participate in drug resistance [23]. Thus, we detected the expression of autophagy markers in MCF-7 cells cocultured with CAF-derived CM under the aforementioned conditions. Compared with the CM from CAFs cultured under normal conditions, the CM derived from GPR30-activated CAFs (wild type GPR30 CAFs pre-stimulated with G1) enhanced the expression of LC3II/I and Beclin1 and decreased expression of p62 in MCF-7 cells (Figure 4A). By contrast, the CM from GPR30-inactivated CAFs (CAFs in which endogenous GPR30 was either antagonized by treatment with G15 or was knockdown by shRNA) did not cause any significant changes in the expression of autophagy-associated proteins (Figure 4A). Interestingly, administration of recombinant human HMGB1 also increased the expression of Beclin1 and LC3II/I proteins and reduced expression of p62 protein in MCF-7 cells (Figure 4B). Furthermore, exhaustion of HMGB1 by neutralizing antibody against HMGB1 notably attenuated these changes in the expression of autophagy-related proteins (Figure 4B). Using LY294002, we inhibited AKT signaling in MCF-7 with the CM before cocultured with TAM. Interestingly, inhibition of AKT signaling attenuated the autophagy induced by CM from G1-treated CAFs in MCF-7 cells. Accordingly, we observed that the autophagosome formation of MCF-7 cells under conditions of TAM administration was increased by stimulation with rHMGB1 and this change was in turn reduced by the use of the neutralizing antibody against HMGB1 (Supplementary Figure 2D, 2E). After inhibition of the PI3K/Akt pathway by LY294002, HMGB1 could not be stimulated by G1, and autophagy was inhibited (Figure 4D). Considering that the TAM-activated GPR30 in CAFs increased the extracellular secretion of HMGB1, these data suggested that activation of GPR30/PI3K/AKT signaling in CAFs could stimulate autophagy in MCF-7 cells through the paracrine action of HMGB1.

**Figure 5:**
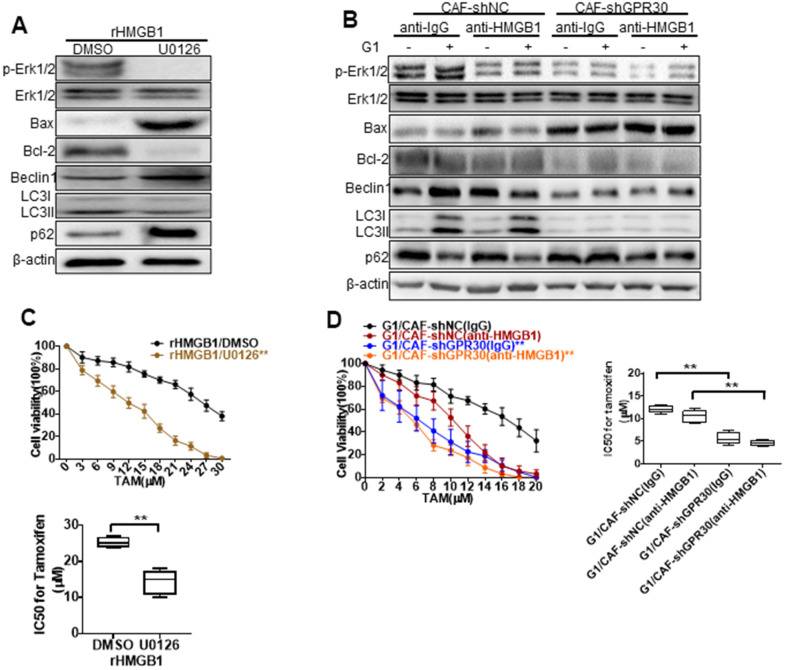
**MEK/ERK is involved in autophagy and promotes apoptosis.** Analysis of autophagy-related proteins (**A**, **B**) and apoptosis markers and cell viability (**C**, **D**) in MCF-7 cells pretreated with rHMGB1 and anti-HMGB1 antibody and U0126 to block ERK signal pathway. β-actin was used as a loading control. All analyses were conducted with 3 different experiments. CAF-shNC (CM): MCF-7 cells cultured with CM from CAF-shNC cells; G1+CAF-sh NC (CM): MCF-7 cells cultured with CM from CAF-shNC cells treated with G1; CAF-shGPR30 (CM): MCF-7 cells cultured with CM from CAF-sh GPR30 cells; G1+CAF-sh GPR30 (CM): MCF-7 cells cultured with CM from CAF-shGPR30 cells treated with G1.

HMGB1-induced autophagy has been ascribed to the activation of the MEK/ERK pathway [22], and thus we aimed to confirm this observation in MCF-7 cells. Indeed, we observed that rHMGB1 stimulated the activation of ERK signaling in tumor cells, accompanied by corresponding changes in the expression of autophagy markers, including enhanced and decreased expression of LC3II and p62, respectively (Figure 4C, Figure 5A). These changes were blocked following administration of an ERK inhibitor (Figure 5A). Moreover, we detected increased phosphorylation of ERK and enhanced autophagy in MCF-7 cells cultured with CM derived from G1-treated CAFs compared with those in MCF-7 cells cultured with CM from CAFs cultured under normal conditions; this change was similar to the effects of rHMGB1 and was significantly attenuated by adding a neutralizing antibody specifically against HMGB1 or U0126 to the CM. However, knockdown of GPR30 or pretreatment of CAFs with G15 reduced the activation of ERK signaling and the effects on autophagy induced by the CM derived from G1-treated CAFs (Figure 5B).

Early research has shown that autophagy can protect cells from the survival impact of chemical agents [24, 25]. We tested the effects of U0126 on the resistance to TAM induced by rHMGB1 in MCF-7 cells, and found that this ERK inhibitor significantly decreased the resistance of cancer cells to TAM, as the IC50 of TAM was decreased from 29 ± 2.828 to 13.67 ± 2.867 (Figure 5C). We thus demonstrated that the MEK/ERK signaling pathway is required for the HMGB1-induced TAM resistance of ERα+ breast cancer cells. As aforementioned, the CM derived from G1-treated CAFs promoted the resistance of MCF-7 cells to TAM. This was similar to the effect of rHMGB1 and was significantly attenuated by adding a neutralizing antibody against HMGB1 (Figure 5D) or U0126 (Figure 5C) to the CM. Meanwhile, knockdown of GPR30 or pretreatment of CAFs with G15 reduced the enhanced resistance to TAM induced by the paracrine action of HMGB1 as well as induced autophagy via MEK/ERK signaling in MCF-7 cells. These data indicated that GPR30-activated CAFs could induce the resistance of MCF-7 cells to TAM through the production of HMGB1.

### GPR30-derived HMGB1 in CAFs promoted breast tumor resistance to TAM *in vivo*

**Figure 6:**
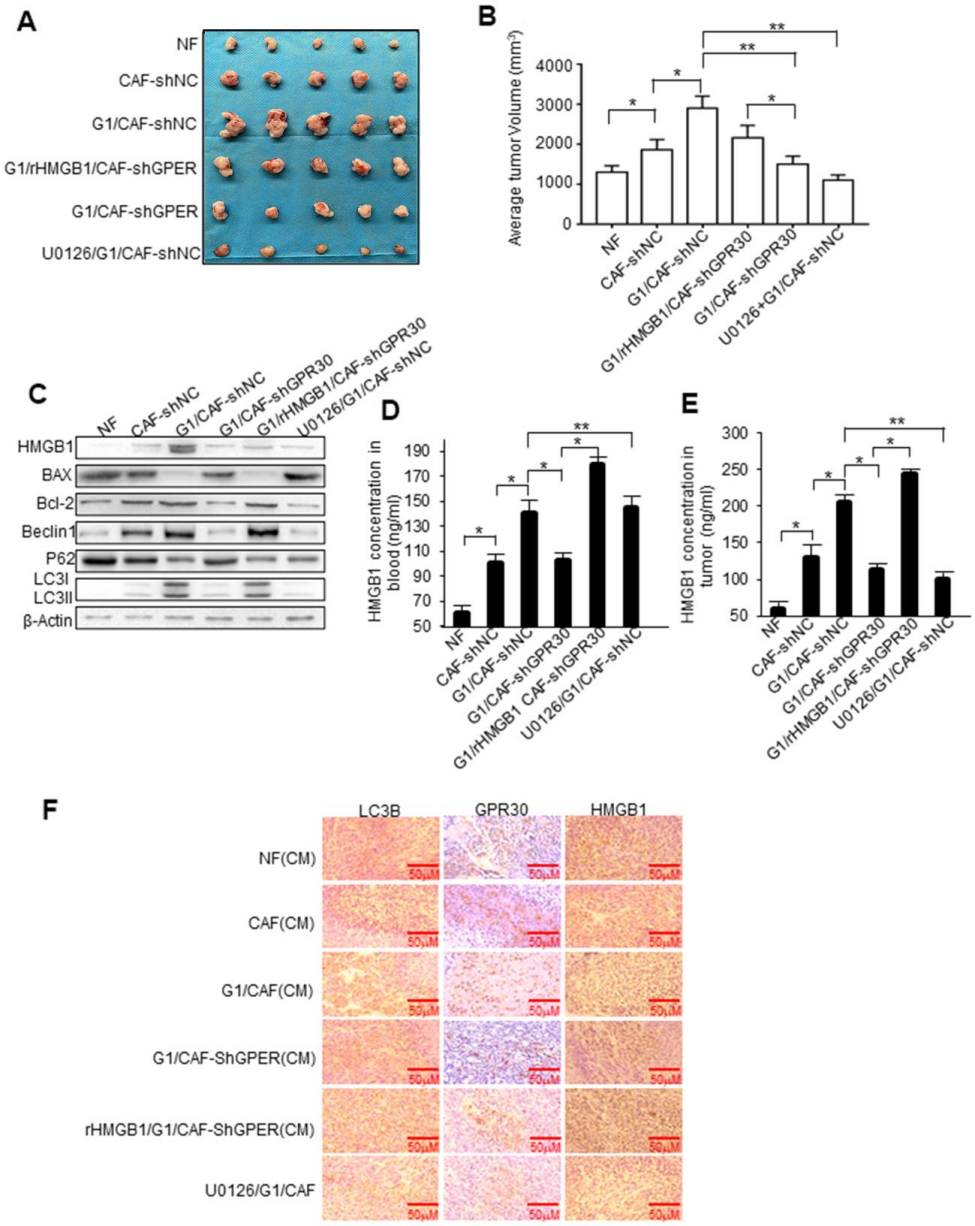
**HMGB1-induced autophagy is required for mammary tumor resistance to tamoxifen in mice.** MCF-7 cells mixed with CAFs or engineered CAFs (CAF/sh-NC, CAF/sh-GPR30) were subcutaneously transplanted into nude mice. Mice were treated with tamoxifen as described in the Materials and Methods. (**A**) Tumor size in mice; mean ± SE of triplicate representative experiments. (**B**) Tumor volume was assessed. (**C**) Autophagy- and apoptosis-related markers in xenografts were quantified by western blotting. U0126 injected into mice at 25 mmol/kg. (**D, E**) The concentration of HMGB1 in xenograft tissues and blood from mice. (**F**) Representative images of LC3B, GPR30, and HMGB1 examined by IHC staining are shown; Scale bar, 50 μm.

To further evaluate the role of GPR30 in CAF-induced resistance of breast cancer cells to TAM *in vivo*, a mixture of MCF-7 cells (5 × 10^6^) and an equal number of CAFs or NFs was injected into the mammary fat pad of mice to establish mouse xenograft models. Tumors in mice injected with MCF-7 and CAFs were larger than those in mice injected with a mixture of MCF-7 and NFs (Figure 6A). The tumor volume of mice with mixed cell lines was pronouncedly significantly larger, whereas knockdown of GPR30 in CAFs attenuated this effect, while with the addition of rHMGB1 can partly rescue tumor volume (Figure 6A, 6B). Treating mice with U0126 (an inhibitor of MEK/ERK) severely blunted G1-stimulated tumor growth in mice injected with MCF-7 and CAFs (Figure 6A, 6B). Furthermore, stimulation with G1 or treatment of mice with rHMGB1 reduced the apoptotic protein BAX and enhancement of BCL2 caused by TAM, while also increasing autophagy, as indicated by the elevation of autophagy-related markers (Figure 6C). Inhibition of MEK/ERK autophagy-associated signaling using U0126 decreased LC3II/I and Beclin1 proteins accompanied by increased and reduced expression of BAX and BCL-2, respectively (Figure 6C). We verified the corresponding levels of HMGB1 in the transplanted tumor tissues and blood in each group of mice (Figure 6D, 6E). Furthermore, immunohistochemistry staining confirmed that the group of mice treated with G1 and injected with the mixed cells, which secreted more HMGB1 and enhanced ability of autophagy as LC3B increasing (Figure 6F). These data supported that activated GPR30-induced expression and secretion of HMGB1 in CAFs contribute to the induction of autophagy in cancer cells to allow them to escape from apoptosis induced by TAM, thereby promoting breast cancer progression.

## DISCUSSION

**Figure 7:**
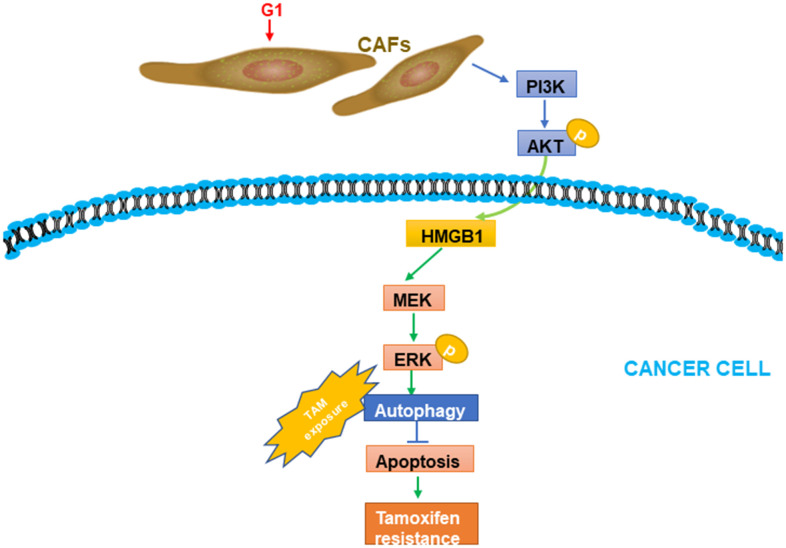
**A working model of GPR30-mediated paracrine effects of HMGB1 on autophagy in CAFs and cancer cells.** G1 stimulation can promote GPR30-induced secretion of HMGB1 by cancer cells; this was proved to be dependent on the PI3K/AKT signaling pathway in CAFs. The elevated HMGB1 induced autophagy, while suppressing apoptosis through MEK/ERK under exposure to tamoxifen.

TAM resistance constitutes a challenge in managing patients with ERα+ breast cancer. The GPR30 protein, which has been detected in not only cancer cells but also CAFs, is an alternative ER that might largely contribute to TAM resistance. Considering the multifaceted role of CAFs in driving the progression of breast cancer [26], we evaluated GPR30-mediated CAF-induced effects on TAM resistance, finding that TAM upregulated the expression and secretion of HMGB1 via GPR30/PI3K/AKT signaling in CAFs. The secreted HMGB1 induced autophagy to enhance the resistance to TAM in MCF-7 cells in an ERK-dependent manner (Figure 7). These results provided novel insights into the underlying mechanism of the resistance to TAM in ERα+ breast cancer cells and a promising strategy to overcome this resistance in patients with breast cancer.

GPR30, the latest identified ER, has attracted increasing attention for its role in resistance to TAM in patients with ERα+ breast cancer [14, 27]. TAM resistance has been linked to an alternative ER, growth factor receptors, and PI3K signaling, among others. Interestingly, GPR30, an alternative ER, was transactivated EGFR and its downstream signaling, including PI3K, and the crosstalk between GPR30 and members of the growth factor family, including EGF, insulin, IGF, and CTGF has also been reported [27, 28]. Moreover, TAM and its metabolite, 4-hydroxy-tamoxifen, both of which are known antagonists of ERα, were demonstrated to function as agonists of GPR30 and promote the proliferation and migration in breast cancer cell lines [14]. Thus, GPR30 was demonstrated to biologically and pharmacologically serve as a predictable driver of resistance to TAM. More importantly, we detected coexpression of GPR30 and ERα in approximately 40 % of the primary breast cancers, with GPR30 constituting a favorable factor for the outcome of patients, but an unfavorable indicator for patients receiving TAM [8, 29]. The expression of GPR30 was firmly correlated with a reduced response rate to primary therapy with TAM in patients with breast cancer [30]. Based on our study and those of other groups, we confidently concluded that GPR30 is an important initiator of the resistance to TAM in ERα+/GPR30+ breast cancers. Similarly, the clinical efficacy of fulvestrant, an agonist of GPR30 and another fundamental agent in the endocrine therapy for patients with ERα+ breast cancer, was also associated with the regulation of GPR30 [31]. Conclusively, GPR30 appears to be a promising target for endocrine therapy, as blocking GPR30 signaling would be an important supplement in endocrine therapy and a strategy to overcome the resistance to endocrine therapy in patients with ERα+ and GPR30+ breast cancer.

Mammary CAFs are the main component of the tumor microenvironment, largely contributing to the development and progression of breast cancer through various factors. Tumor progression, including recurrence and even distant metastasis during the administration of TAM, is known to indicate resistance to TAM [32]. Accordingly, CD146-negative CAFs promoted the resistance of ERα+ breast tumors to TAM [33]. Likewise, CAFs derived from clinical-luminal breast cancers induced resistance to TAM by decreasing the levels of ERα when cultured with MCF7 and T47D luminal BRCA cell lines in both *in vitro* and *in vivo* experiments [34]. Intriguingly, GPR30 protein has been repeatedly detected in CAFs from breast cancer patients [6, 15]. Moreover, TAM, in addition to estradiol, promoted the proliferation and migration of CAFs via GPR30 [15]. Thus, GPR30-positive CAFs might probably act as natural inducers of resistance to TAM in patients with breast cancer. Further, we and other groups demonstrated that GPR30 upregulated the expression of aromatase to sustain the resistance of breast cancer cells to TAM [11]. Moreover, we also confirmed that cytoplasmic GPR30 translocation in CAFs mediated the cAMP/PKA/CREB/glycolytic axis to confer resistance to TAM in ERα+ breast cancer cells [6]. In the present study, we found that GPR30 activated in CAFs induced the expression and secretion of HMGB1, which conferred resistance to TAM in MCF-7 cells. These results provided novel insights into the contribution of both CAFs and GPR30 to resistance to TAM. Prospectively, CAFs might be considered to be a better target for breast cancer treatment, as genomic stability in CAFs is higher than that in tumor cells. Considering that GPR30 was detected in CAFs in approximately 40% of the primary breast cancer tissues and was significantly associated with the expression of ERα+ in solid tumors [15], the GPR30 protein in CAFs might be the key to overcome the resistance to TAM in these patients.

The GPR30 protein triggers transcriptional regulation and a series of rapid signaling events in various cellular backgrounds [35, 36]. Using microarrays, the specific response of a number of genes to the activation of GPR30 has been identified in breast cancer cells [36]. In these arrays, genes fulfilling the following criteria: significantly upregulated/downregulated by agonists of GPR30 (including E2, G1, and TAM) and reduction of this positive response by an antagonist of GPR30 (G15/G36) or antisense-mediated GPR30 knockdown, were considered as potential GPR30 target genes [36]. Using mRNA microarrays in the present study, we identified 165 genes regulated by the activation of GPR30; these genes were involved in various signaling pathways. To the best of our knowledge, our study went a step further to provide additional information on the global estrogenic transcriptional actions mediated by GPR30 that have been reported in breast CAFs so far [17]. In addition, coordination/activation of GPR30 triggers downstream cascades, including ERK1/2/AKT or MAPK/PI3K/AKT signaling, both of which have been recognized as rapid estrogenic effects [17, 37]. Herein, we confirmed that the upregulation of HMGB1 induced by TAM in CAFs was dependent on GPR30/PI3K/AKT signaling; thus, we assumed that these transcriptional effects mediated by GPR30 were actually the downstream events of rapid signaling. Moreover, the extracellular secretion of HMGB1 was also demonstrated to be increased in response to TAM administration. Considering the nature of HMGB1 as a cytokine and the paracrine model by which CAFs might drive the progression of breast cancer, we further demonstrated that activation of GPR30 in CAFs induced resistance to TAM in MCF-7 cells through the paracrine action of HMGB1. These data have provided an in-depth understanding into the diversity of GPR30-mediated estrogenic effects in the breast cancer microenvironment and the GPR30-mediated and CAF-dependent mechanism of TAM resistance. However, further studies are needed to understand the complexity of these transcriptional effects mediated by GPR30.

So far, we know that drug resistance is linked to enhancement of growth, anti-apoptosis, or cell survival processes. However, increasing evidence has supported that the process of autophagy might also contribute to the multidrug resistance of breast cancer cells [3, 23, 38]. We found that GPR30-mediated autophagy could reduce apoptosis, and thus result in breast cancer cell resistance to TAM, but this process could be mitigated by interruption of autophagy, in accordance with previous studies [22, 24]. Increased degradative autophagy was reported to boost the renewal of damaged organelles and proteins, thus promoting cancer cell survival by facilitating resistance to therapy [39]. With accumulating evidence shedding light on autophagy mediators, we found this result to be true for a plethora of factors ranging from cytokines, to starvation, host factors, specific genetic or epigenetic alterations, and even viral particles [40]. We identified HMGB1 to be autophagy-modulated in a paracrine manner, consistent with previous observations that autophagy controlled the secretion of such factors in other systems [41]. HMGB1 is systemically elevated in CAFs from luminal breast cancer [7], and thus has been associated with resistance to endocrine therapy. In addition, it is known to be secreted from stromal fibroblasts found in a number of cancer types [42]. Therefore, HMGB1-induced autophagy might be a promising landmark in tumor growth and for the progression of endocrine therapies for breast cancer.

In this study, we delineated an interaction by which CAFs might contribute to the TAM-acquired resistance of breast cancer cells via the paracrine action of HMGB1. Notably, this interaction was demonstrated to be initiated by CAF-expressed GPR30, a well-known alternative ER largely contributing to resistance to TAM. Mechanistically, this interaction was found to be dependent on transcriptional regulation mediated by GPR30/PI3K/AKT signaling in CAFs and MEK/ERK signaling-induced autophagy in ERα+ breast cancer cells. We could thus conclude that GPR30, an initiator, induced the resistance of cancer cells to TAM not only directly but also in a CAF-dependent manner. Prospectively, targeting GPR30 and its associated cascade should be an effective strategy to overcome the resistance to TAM and to supplement endocrine therapy in patients with ERα+ breast cancer. Clinical detection of GPR30, both in the tumor core and stroma, would probably benefit these patients. Therefore, we expect that the guidelines for breast cancer would be revised to capture the role of GPR30 in the future.

In summary, we found that activated GPR30 in CAFs induced the accumulation of HMGB1, which enhanced resistance to TAM in breast cancer cells via a paracrine effect. However, knockdown GPR30 or pretreatment of CAFs with G15 reduced the enhanced resistance to TAM induced by the paracrine action of HMGB1, which eventually induced autophagy via MEK/ERK signaling in MCF-7 cells. Our study has identified a novel treatment for overcoming endocrine resistance by targeting GPR30.

## MATERIALS AND METHODS

### Clinical samples

### Cell culture

CAFs and NFs were isolated from breast tumor and their paired normal tissues, identified using CAF-related biomarkers, and immortalized using the human telomerase reverse transcriptase gene (hTERT), as previously described [43]. MCF-7 cells were cultured in DMEM phenol red-free medium supplemented with 10 % fetal bovine serum (Gibco, Australia). Cells were cultivated in an incubator at 37° C in 5 % CO_2_. The GPR30-shRNA and NC-shRNA constructs were inserted into the LV3 lentiviral vector, respectively, and the lentivirus stably carrying GPR30-shRNA was harvested after continuous exposure to 9 μg/mL puromycin for 7~14 days [11]. The GPR30-shRNA lentiviral expression vector was obtained from Gene Pharma (Shanghai, China), The sequence of GPR30 shRNA was 5’-CGCTCCCTGCAAGCAGTCT-3’. Paracancerous or fibrous tissue was isolated from patients who had undergone surgery. In addition, nontumor-associated fibroblasts were collected for each case from adjacent uninvolved breast tissue at the First Affiliated Hospital of Chongqing Medical University.

### Isolation and culture of primary fibroblasts

Fibroblast contraction assay was performed according to the published method [44, 45]. Tissues were washed 3 times with sterile PBS containing with antibiotics (100 U/ml penicillin, 100 μg/ml streptomycin and 50 μg/ml gentamycin). The tissues were minced and digested with 0.1% collagenase type I (C0130, Sigma, Saint Louis, MO, USA) at 37° C for 8–12 h [44], tissues were carefully pipetted up and down for a couple of times using culture medium. The mixtures were centrifuged and washed with DMEM to remove the fat and tissue debris. Then, the mammary tissues were cultured in DMEM with 10% fetal bovine serum (FBS, Gibco, Australia) for about two days. The most adherent cells isolated from tumor tissues were named “CAFs”, and from paracancerous normal tissues named “NFs”. Cell purity was identified by immunohistochemistry for Fibronectin, α-SMA and FAP [46].

### Reagents

The following reagents were used: rHMGB1 (Cat No. #1690-HMB-050, R&D Systems, Minneapolis, MN, USA), G1 (Cat. No. 3577/10, TOCRIS, Bioscience, Bristol, UK), G15 (Cat. No. 3678/10, TOCRIS, Bioscience, Bristol, UK). E2, TAM, were obtained from Sigma–Aldrich (St. Louis, MO, USA). U0126, and the LY294002 were purchased from Millipore (Temecula, CA, USA). E2 was dissolved in ethanol and other drugs were solubilized in dimethyl sulfoxide (DMSO; Sigma–Aldrich). The following antibodies were used: anti-HMGB1 neutralizing antibody (Cat No. H00003146-M08, R&D systems, Minneapolis, MN, USA); p-ERK1/2 (Cat No. AP0484P, diluted 1:1000), ERK1/2 (Cat No. BS90472, diluted 1:1000), Akt (Cat No. BS6473, diluted 1:1000) were purchased from Bioworld (St Louis Park, MN, USA). p-Akt (Cat No. 4060, Cell signaling Technology, USA, diluted 1:1000), LC3B (Cat No. 2775 Cell Signaling Technology, diluted 1:1000), P62 (Cat No. 5114, Cell Signaling Technology, diluted 1:1000); Beclin1(Cat No. 210498, diluted 1:1000), GPR30 (Cat No. ab39142, diluted 1:250), HMGB1 (Cat No. ab18256, diluted 1:500), Bcl-2 (Cat No.32124, diluted 1:1000), BAX (Cat No. 182734, diluted 1:1000) were purchased from Abcam (Cambridge, MA, USA), and β-actin (Cat No. TA-09, diluted 1:1000) from Zhongshan Golden Bridge (Beijing, China).

### Western blotting analysis

Proteins from breast cancer tissues or cell lines were harvested using RIPA protein extraction buffer (Boster, China) with protease inhibitor (Beyotime, Shanghai, China) and separated using SDS-PAGE. The BCA Protein Assay Kit (ThermoFisher Scientific, Waltham, MA, USA) was used to measure the concentration of proteins, and 20 μg of total protein, mixed with 1 × SDS loading buffer, was loaded per lane. Proteins of lysates were separated by 10 % SDS- PAGE and transferred to polyvinylidene difluoride (PVDF) membranes (ThermoFisher Scientific). Membranes were blocked for 1 h with 5 % skim milk at room temperature. PVDF membranes were then incubated with primary antibodies at 4° C overnight, followed by incubation with the secondary antibodies for 2 h at 37° C. The indicated proteins were visualized using the enhanced chemiluminescence system (Amersham, Freiburg, Germany).

### Quantitative RT-PCR analysis

Total RNA was extracted from MCF-7 cells and breast cancer tissues using Trizol (TaKaRa, Dalian, China) following the manufacturer’s instructions. The OD260/280 and OD260/230 values were measured to ensure the quality of extracted RNA. Then, cDNA was synthesized from 1 μg of total RNA using a reverse transcriptase kit (Takara, Japan). qRT-PCR with the Power SYBR Green qPCR Super Mix-UDG (Invitrogen, NY, USA) was performed to quantify the expression level of HMGB1 on a BIO-RAD system. β-actin was used as an internal control. The expression of HMGB1 was normalized to that of β-actin, and quantified according to the 2-^ΔΔCT^ method. Primer sequences were GPR30 (Forward TGGGGAAGAGGCCACCA; Reverse: CGTGGAGCTGCTCACTCTCTG), HMGB1 (Forward: CACTGGGCGACTCTGTGCCTCG; Reverse: CGGGCCTTGTCCGCTTTTGCCA), and β-actin (Forward: CGCGAGAAGATGACCCAGAT; Reverse: GGGCATACCCCTCGTAGATG).

### CM collection

CAFs and NFs (2.0 × 10^6^) were seeded into a 6 well plate in in serum-free DMEM (Gibco, USA) growth phenol red-free medium and cultured for 12 h to reach 70~80 % confluency, and then incubated in DMEM with 0.5 % FBS. Subsequently, the CM was collected in addition to fresh complete phenol red-free medium and then stored at -80 or -20° C until use.

### ELISA

HMGB1 protein expression in CM was evaluated using an ELISA test kit (NOVAS) according to the manufacturer’s instructions. Using the respective ELISA software, a standard curve was prepared using the absorbance and concentration values of wells with standards, and the values for the concentration of HMGB1 in the supernatant of CAF-shNC, CAF-shGPR30, G1 + CAF-shNC, and G1 + CAF-shGPR30 group of cells were calculated accordingly. The experiment was repeated 3 times. The evaluation of the concentration of HMGB1 in tumors collected from mice followed the same procedure.

### Measurement of intracellular Ca^2+^ mobilization

We measured the intracellular Ca^2+^ mobilization using the Ca^2+^-sensitive fluorescent probe marked with Fluo-3/AM (1-[2-amino-5-(2,7-dichloro-6-hydroxy-3-oxo-9-xanthenyl) phenoxy]-2-(2-amino-5-methylphenoxy)-ethane-N,N,N0,N0-tetraacetic acid, penta acetoxymethylester) (Beyotime). Detailed steps of the procedure were provided in our previous study [15].

### Cell viability assay

Cells were seeded in 96-well plates at the density of 1 × 10^4^ cells per well, treated with different conditional media 8 h, and then replaced with phenol red-free medium for another 24h before the addition of G1, G15, TAM, U0126 at the designated concentrations. The final concentration of vehicle (DMSO) was 0.1 %. At the end of treatment, cells were incubated with 10 μL of Cell Counting Kit-8 solution (Boster) for 1 h at 37° C, and then a digital spectrophotometer was used to measure the optical density (OD) 450 value, which was expressed as a percentage (%) of the value of the control.

### Immunohistochemical (IHC) and immunofluorescence (IF) staining

Cells were planted at a density of 1 × 10^5^ cells per well on glass coverslips for 24 h, fixed with 4 % paraformaldehyde, penetrated with 0.1 % Triton, and briefly blocked with 5 % goat serum at 25° C. Cells were then incubated overnight at 4° C with primary antibodies targeting LC3B (all dilutions were 1:200). After washing with PBS, cells were stained with a FITC-labeled goat anti-rabbit secondary antibody (1:100; Zhongshan Golden Bridge, Beijing, China) for 10 min, and with 4’,6-diamidino-2-phenylindole (DAPI) for 5 min.

Commercial rabbit anti-GPR30 and anti-HMGB1 polyclonal antibodies (Abcam) were used for immunohistochemical staining, as previously described [9]. Nonspecific binding was blocked by incubation in 5 % goat serum solution for 30 min at 37° C. Slides were exposed to primary antibody for 2 h at 37° C. Sections were incubated with HRP-conjugated goat anti-rabbit IgG for 20 min at 37° C. GPR30/HMGB1-staining was considered positive when distinct staining of at least 10 % of fibroblasts was observed [47]. IHC and IF images were captured using a Nikon Eclipse 80i microscope (Nikon, Tokyo, Japan). The staining intensity and percentage of positive tumor cells of each section were calculated using the immunoreactive score (IRS) [29]. Staining patterns were classified as low (IRS: 0–4) and high (IRS: 6–12) protein expression of GPR30 or HMGB1.

### Specimens

Archived paraffin-embedded samples of breast cancer were obtained from the Clinical Diagnostic Pathology Center, Chongqing Medical University (Chongqing, China). All samples were from patients who had undergone surgery at the 1^st^ Affiliated Hospital of Chongqing Medical University from 2000 to 2017 and were diagnosed in the same center. The validation cohort tissue microarray (TMA) included 40 cases of tissues from recurrence sites and 25 paranoncancerous tissues. The detailed clinical information of each individual specimen was pathologically confirmed. The experiments were approved by the Ethics Committee of the First Affiliated Hospital of Chongqing Medical University.

### Xenografts

Xenograft models were established in 4~5 week-old female nude mice by implanting 5 × 10^6^ cells into their mammary fat pads. Mice were kept under sterile conditions and receiving sterile nutrition and water (Animal Experimental Center of Chongqing Medical University, Chongqing, China). Mice were randomly assigned to experimental groups at n = 5 per group. All the procedures involving animals and their care were conducted in conformity with institutional guidelines. Slow-release estradiol pellets (Innovative Research of America, Sarasota, FL, USA; 0.3 mg) were subcutaneously implanted into the dorsal flank of mice [48]. TAM and G1 were dissolved and diluted to their proper concentrations using absolute ethanol. U0126 was diluted in DMSO at 10 mmol/L as a stock solution. We administered 25 and 50 mmol/kg U0126 to animals through weekly intraperitoneal (i.p.) injections. Mice were administered a subcutaneous injection (0.1 mL/mouse) of TAM (50 μg) after 4 weeks, and G1 (4 μg) or G15 (4mg) once daily after 3 weeks. Tumor size was measured using a caliper according to the following formula: volume = (width)^2^ * length/2 (Figure 5A, 5B). An endpoint of 2.0-3.0 cm^3^ was adopted for tumor size.

### Response of breast cancer cells treated with recombinant HMGB1 to TAM

MCF-7 cells were seeded in 96-well plates at 5000 cells per well and cultured overnight in DMEM +10 % FBS. Cells in triplicate wells were then treated with 100 ng/mL and 500 ng/mL rHMGB1 in the presence or absence of 10 mg/mL HMGB1-neutralizing antibody (anti-HMGB1) and then treated with TAM. Cell viability was measured at OD = 450 mm 24 h later using CCK8 (Boster).

### Statistical analysis

All experiments were performed at least 3 times, and statistical analyses were performed using SPSS 22.0 software. For TMA, the difference between cancer tissues and paired paranoncancerous tissues was estimated using the χ2 test. The correlation between GPR30 and HMGB1 was estimated by Spearman's correlation analysis. The association between HMGB1 and GPR30 staining and the clinicopathologic parameters of patients with TAM resistance was evaluated using the χ2 test. All values are shown as means ± standard deviation (SD). Two-group comparisons were performed using the Student’s *t*-test. P < 0.05 was considered statistically significant. The overall survival rate or the relapse-free survival rate of patients with breast cancer was estimated using Kaplan–Meier analysis.

## Supplementary Material

Supplementary Figures
