# Supplementary material for: GPR30-mediated HMGB1 upregulation in CAFs induces autophagy and tamoxifen resistance in ERα-positive breast cancer cells
Source: Aging (Albany NY). 2021 Jun 28;13(12):16178–97. doi: 10.18632/aging.203145 (PMC8266353; doi:10.18632/aging.203145)
Supplement: Supplementary Figures [file aging-13-203145-s001.pdf]

SUPPLEMENTARY FIGURES

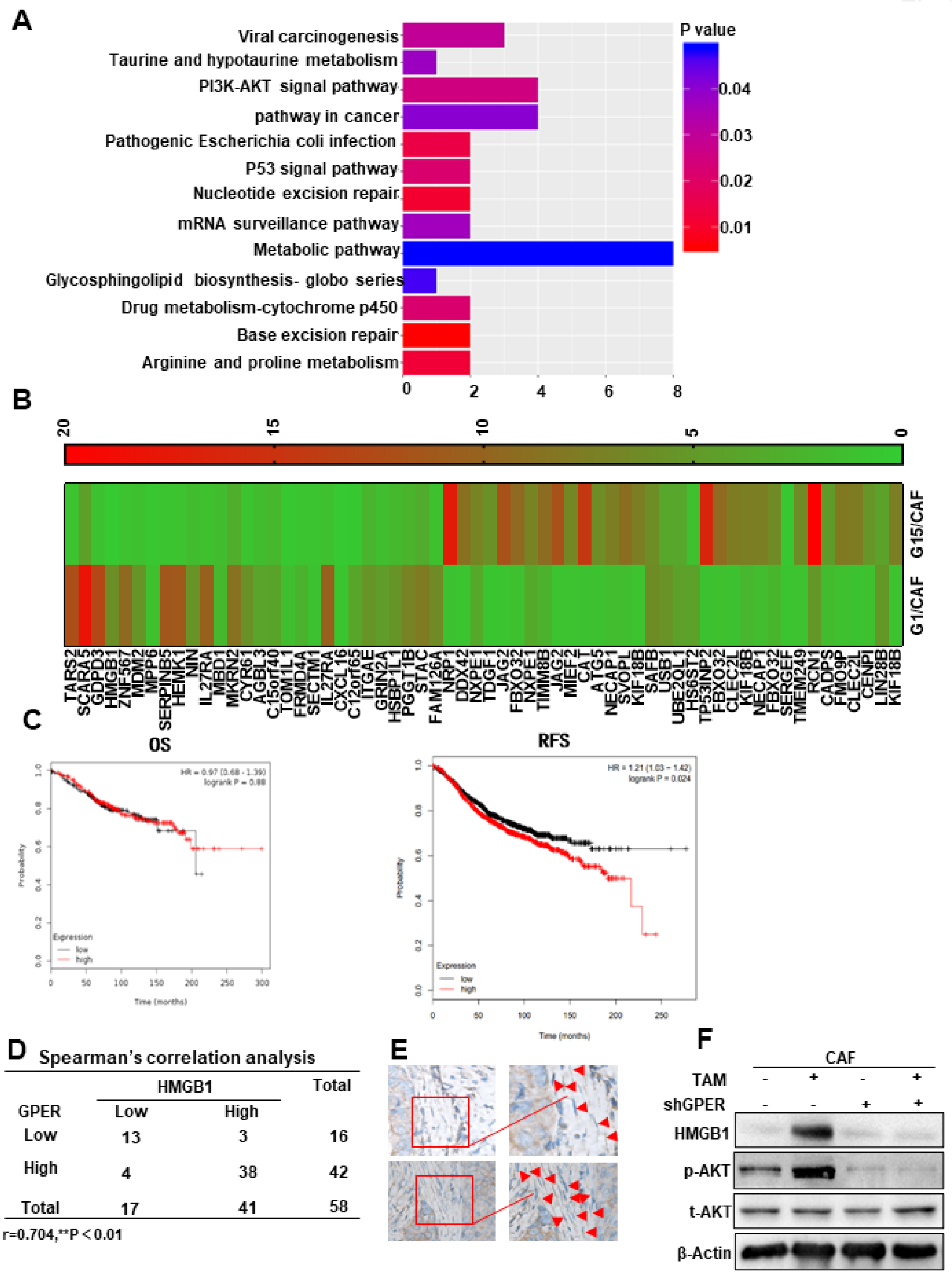

Supplementary Figure 1. Related to Figure 2: HMGB1 expression in mRNA microarrays and its clinical significance. (**A**) Heat-map of changed genes and cytokines in the G1 group compared with those in the group administered the combination of TAM and G15. Red and green indicate up-and downregulated genes, respectively. Fold changes>1.5; $P < 0.05$, ctrl group: CAF cells without any treatment; TAM/G15 group: CAF cells pretreated with G15 for 30 min, and then treated with TAM; G1 group: CAF cells pretreated with G1 for 30 min. TAM group: CAF cells treated with TAM; $^{**}P < 0.01$, (n = 3). (**B**) KEGG signaling pathway prediction. (**C**) Online prediction of overall survival (OS) and relapse-free survival (RFS) of patients with breast cancer. Database: http://kmplot.com/analysis/. (**D**) Correlation between GPR30 and HMGB1 in ER-positive breast cancer tissues using SPSS22.0. (**E**) IHC for HMGB1 in serial sections of highly expressing GPR30 tissues. (**F**) Phosphorylation of AKT was altered under stimulation with TAM.

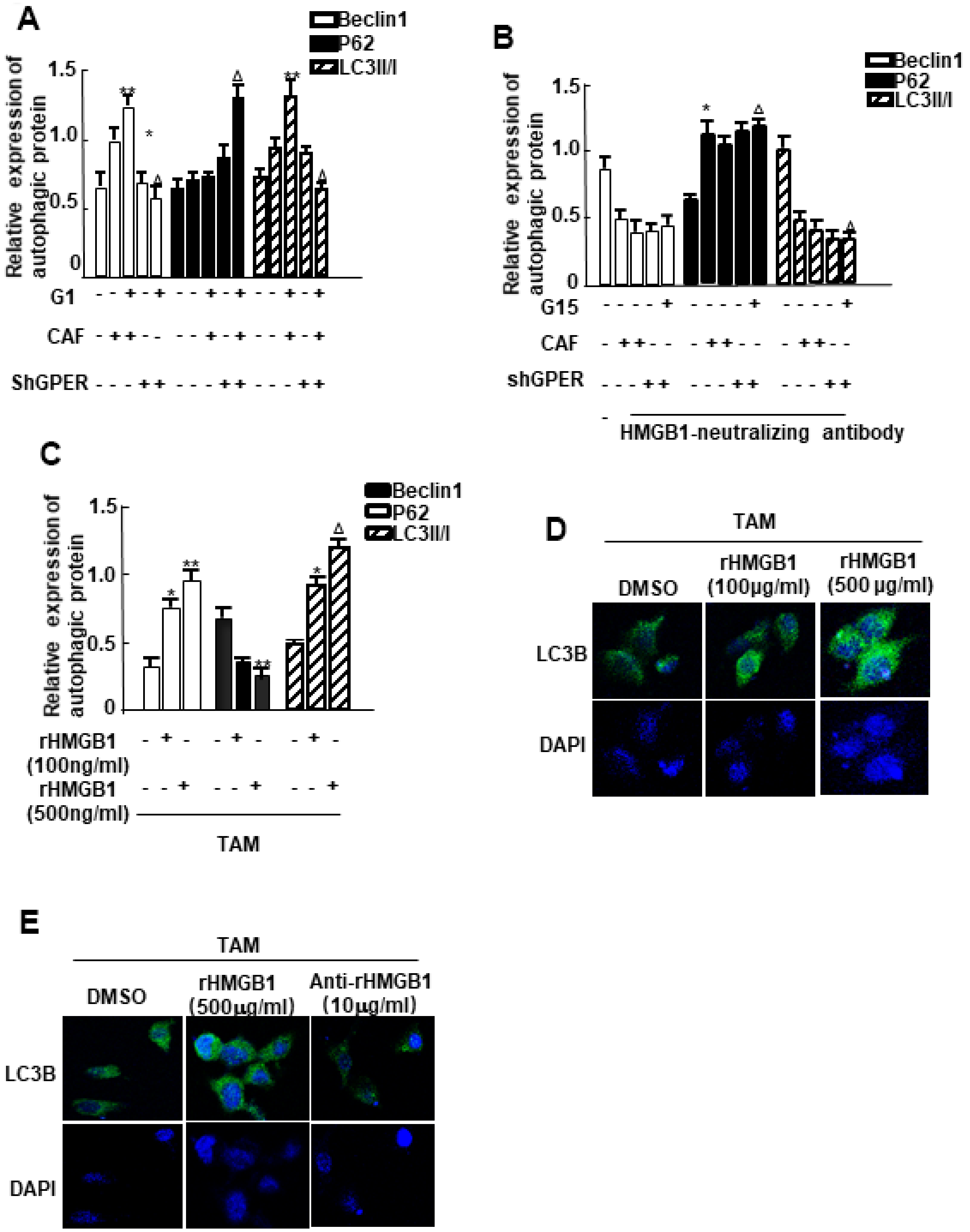

Supplementary Figure 2. Related to Figure 4: Quantified data by Quantity one software and autophagosome formation. (**A**–**C**) Quantification of the data of western blotting using the Quantity one software. (**D**, **E**) mGFP-LC3B expression vectors were transfected into MCF-7 cells cocultured with recombinant HMGB1 or anti-HMGB1 antibody. The numbers of LC3B puncta were evaluated under a fluorescence microscope.
